# Up‐regulation of CDHR5 expression promotes malignant phenotype of pancreatic ductal adenocarcinoma

**DOI:** 10.1111/jcmm.15856

**Published:** 2020-10-06

**Authors:** Junyi Gao, Mengyi Wang, Tong Li, Qiaofei Liu, Lei You, Quan Liao

**Affiliations:** ^1^ Department of General Surgery Peking Union Medical College Hospital (PUMCH) Chinese Academy of Medical Sciences & Peking Union Medical College (CAMS & PUMC) Beijing China

**Keywords:** CDHR5, isoform, pancreatic cancer, prognosis

## Abstract

CDHR5 has been reported to play key roles in carcinogenesis of various cancers, but its roles in pancreatic cancer have not been reported. The present study was designed to investigate its clinical value in pancreatic ductal adenocarcinoma (PDAC). Tissue microarray‐based immunohistochemistry was performed to analyse the correlation between CDHR5 expression and clinical and pathological features of PDAC, as well as the CDHR5 expression during tumour progression. Cell function assays were performed to investigate CDHR5’s effects on PDAC cells. Moreover, qRT‐PCR was applied to investigate the expression of CDHR5 isoforms in PDAC cells. Expression of CDHR5 was higher on the membrane of PDAC cells. This high expression level was associated with shorter overall survival of PDAC patients and was identified as an independent prognostic factor for overall survival by multivariate Cox regression analysis. In addition, expression level of CDHR5 presented an increased trend in the occurrence and progression of PDAC. Cell experiment suggested that CDHR5 could notably promote invasion and migration of PDAC cells. Moreover, analysis of CDHR5 isoforms indicated CDHR5‐L was the major isoform expressed in PDAC cell lines. CDHR5 appears to be a promising and novel prognostic factor for PDAC, and its promotion in PDAC metastasis might be ascribed to the isoform CDHR5‐L.

## 1 INTRODUCTION

Pancreatic ductal adenocarcinoma (PDAC) is the forth lethal malignancy, and its overall 5‐year survival rate is only 3%‐9%.^1^, ^2^, ^3^ Surgical resection is currently the only potential curative option, but over 80% of PDAC patients have suffered an unresectable tumour at the time of diagnosis, in which most of the tumours have been locally advanced invading or even occurred metastasis.^4^ Although lots of efforts have been invested in researching adjuvant and neoadjuvant therapy for pancreatic cancer, its indications and benefits are still controversial and need further studies.^5^, ^6^ Therefore, it is vital to search novel molecular targets for inhibition of tumour metastasis.

*CDHR5*, also known as *MLPCDH*, *MUCDHL*, *MUPCDH* and *MU‐PCDH*, is a member of the cadherin superfamily and encodes the transmembrane protein CDHR5. CDHR5 contains four main functional domains, including the cadherin domain, the mucin‐like domain, the proline‐rich region and the PDZ binding motif. In normal physiological conditions, CDHR5 is generally distributed in the surface of epithelial cell of kidneys, lungs and other organs, playing an essential role in various cellular process including cell adhesion and branching morphogenesis of organs.^7^ Studies on renal cell carcinoma, colorectal cancers and liver cancers revealed the low expression of CDHR5 on the surface of tumour cells and its correlation with poor prognosis.^8^, ^9^, ^10^ However, the roles of CDHR5 in pancreatic cancer have not been reported.

In this study, tissue microarrays were used to investigate the relationship between CDHR5 expression and clinicopathological characteristics of PDAC patients. In addition, cellular and molecular studies have been applied to further explored the roles of CDHR5 in pancreatic cancer cells and the potential mechanisms.

## 2 MATERIALS AND METHODS

### 2.1 Tissue microarrays

Tissue microarrays (TMA) used in this study, the HPan‐Ade170Sur‐01 and BIC14011, were obtained from the US Biomax (https://www.biomax.us). The HPan‐Ade170Sur‐01 contained 170 cores from 71 pancreatic cancer cases with tumour and matched normal adjacent tissue (NAT) and 28 cases with tumour only. Follow‐up information for corresponding cases was collected, including age, gender, tumour size, tumour site, pathological grade, TNM stage, nerve invasion and survival time. The TNM stage was reinterpreted according to the 8th edition Staging Manual of the American Joint Committee on Cancer (AJCC). The BIC14011 contained 24 cases/48 cores, including chronic pancreatitis, pancreas intraepithelial neoplasia (PanIN) and PDAC. Histological diagnosis of all the cases above was confirmed by one attending pathologist.

### 2.2 Immunohistochemistry staining and evaluation

The immunohistochemistry (IHC) staining was performed on the TMAs. Both TMAs were deparaffinized with xylene and rehydrated in a series of ethanol solutions decreasing in concentration. They were then undergoing antigen retrieve step in Colloidal Bismuth Subcitrate buffer solution pH 6, followed by treated with 3% H_2_O_2_‐methanol for 10 minutes. After blocking the non‐specific antigens with 5% goat serum, TMAs were incubated with polyclonal anti‐MUPCDH antibody (ab121306, Abcam; 1:100) overnight at room temperature. At the end of the reaction, the TMAs were reacted with 3,3′‐diaminobenzidine, tetrahydrochloride (DAB) and counterstained with haematoxylin.

Evaluation of the staining results was finally carried out independently by two pathologists. The CDHR5 immunoreactivity was semi‐quantitatively scored, based on fraction of positive tumour cells and staining intensities: The fraction of positive tumour cells was scored as 1 (0%‐24%), 2 (25%‐49%), 3 (50%‐74%) or 4 (75%‐100%), and the stain intensity was recorded as score 0 (negative), 1 (weak staining), 2 (medium staining) or 3 (heavy staining). As a result, the total IHC score was calculated by multiplying both scores (fraction of positive cells × stain intensity, range 0‐12).

### 2.3 Patients and study design

HPan‐Ade170Sur‐01 was applied to analyse the expression of CDHR5 in PDAC and NAT, as well as the relationship between CDHR5 expression and patients’ clinicopathological characteristics. To investigate the difference of CDHR5 expression between tumour and para‐tumour tissues, patients were included according to the following criteria: with both PDAC tissue and matched NAT; without pre‐operative chemotherapy or radiotherapy; pathological diagnosis as PDAC. To further study the relationship between CDHR5 expression and patients’ clinicopathological characteristics, patients were included according to the following criteria: less than 75 years old; without pre‐operative chemotherapy or radiotherapy; pathological diagnosis as PDAC; and without perioperative death.

BIC14011 was used to detect the potential changes of CDHR5 expression during tumour progression, and patients were included according to the following criteria: without pre‐operative chemotherapy or radiotherapy; pathological diagnosis as pancreatitis, pancreas intraepithelial neoplasia or PDAC.

### 2.4 Cell lines and culture conditions

AsPC‐1, BXPC‐3, Panc‐1 and MIA PaCa‐2 cell lines were gifted from Heidelberg University. The AsPC‐1 and BXPC‐3 were cultured in RPMI1640, and the MIA PaCa‐2 and Panc‐1 were grown in Dulbecco's modified Eagle/High Glucose (DMEM/High Glucose). Both culture mediums were supplemented with 10% heat‐inactivated foetal bovine serum (SH30084.03, HyClone), and all the cells were cultured at 37°C under a humidified 5% CO_2_ atmosphere.

### 2.5 Western blot

Proteins were extracted from cells by RIPA buffer (1% Triton X‐100, 0.1% sodium dodecyl sulphate, 1% sodium deoxycholate, 0.15 m NaCl and 10 mm Tris, pH 7.2) with a protease inhibitor cocktail Set III (539134, Merck). Western blotting was performed according to the standard procedures. The polyvinylidene fluoride (PVDF) membrane was incubated in primary antibodies CDHR5 (SC‐166953, Santa Cruz; 1:1000), GAPDH (H‐12, Santa Cruz; 1:1000) or β‐actin (4970S, Cell Signaling Technology; 1:1000) overnight at 4°C and then in secondary antibodies goat antimouse IgG or goat anti‐rabbit IgG (C1308 and C1309, Applygen; 1:10 000) at room temperature for about 1 hour. Western blot signals were detected by using Immobilon Western Chemiluminescent HRP Substrate (WBKLS0050).

### 2.6 Cell transfection

Experiments of mRNA silencing were conducted by transfecting siRNA oligonucleotides directed against CDHR5 into AsPC‐1 cells with Lipofectamine™ 3000 Transfection Reagent (Invitrogen). The target sequence of siRNA‐CDHR5: GCCATCACATATCGAATTA. CDHR5 overexpression plasmids were constructed and used for the transfection with Lipofectamine™ 2000 reagent (Invitrogen).

### 2.7 RNA extraction and quantitative real‐time PCR

Total RNA was extracted from several PDAC cell lines by using the TRIzol reagent according to the manufacturer's instructions. To obtain the cDNA for quantitative real‐time PCR (qRT‐PCR), 1000ng of total RNA was reverse‐transcribed using the PrimeScriptTMRT reagent Kit (RR037A, TAKARA). And the qRT‐PCR was then performed with the GoTaq^®^ qPCR Master Mix (SYBR). β‐actin household gene was chosen to normalize the results, and primers for CDHR5, CDHR5‐L and CDHR5‐M were listed as follows:

CDHR5‐forward: 5′‐ACCCAGCTAAGGGTGTTCGT‐3′
CDHR5‐reverse: 5′‐TGGCACCTGCTGTCATTTCC‐3′
CDHR5‐L‐forward: 5′‐CTCCCACCAACCAACCAC‐3′
CDHR5‐L‐reverse: 5′‐CATATCCACCACCGAGAAGC‐3′
CDHR5‐M‐forward: 5′‐TGGAGGGAGAGGTTGTGCT‐3′
CDHR5‐M‐reverse: 5′‐GGCCGCCACCTGTGGAGG‐3′

### 2.8 Cell proliferation assay

Forty‐four hours after siRNA transfection, cells were collected and plated in 96‐well plates at a density of 5000 cells/100 μL/well. The Cell Counting Kit‐8 (CK04, Dojindo) was applied to analyse cell proliferation on 0, 24, 48, 72 and 96 hours. After 3‐hour incubation with CCK‐8 reagent, cell viability was analysed by detecting the absorbance at 450 and 630 nm.

### 2.9 Transwell assay

Transwell assays were performed to test cell migration and invasion abilities. The 24‐well transwell plates used in this assay contained a polycarbonate membrane with 8.0‐μm pores. In the invasion assays, chambers were pre‐coated with 10‐fold diluted Corning Matrigel. Cells transfected by siRNAs were seeded onto the upper surface of the transwell membrane at a density of 30 000 cells/100 μL/well. After 48 hours, we gently cleared cell in the upper surface of chambers and stained cells migrating to the bottom with haematoxylin and eosin. Cell counting was performed in 5 randomly fields under light microscope.

### 2.10 Statistical analysis

Compared *t* test was applied to analyse the difference of CDHR5 expression between PDAC tissue and adjacent normal ductal tissues. Chi‐square test and Fisher's exact test were used to evaluate significance of associations between CDHR5 expression and clinicopathological parameters. Survival analyses were conducted by using uni‐ and multivariate Cox proportional hazards and Kaplan‐Meier analyses. To define the expression status of CDHR5, ROC curve and median method were used to determine the cut‐off value. Our study was carried out under the guideline of medical ethics committee at Peking Union Medical College Hospital, and all the experiments above were repeated for three times, and results were presented as mean ± SEM values. The statistical analyses were performed with SPSS for mac, version 24. All statistical tests were two‐sided, and the significance level was defined as *P* < .05.

## 3 RESULTS

### 3.1 Immunohistochemical evaluation of CDHR5 in PDAC and adjacent normal tissues

**Figure 1:**
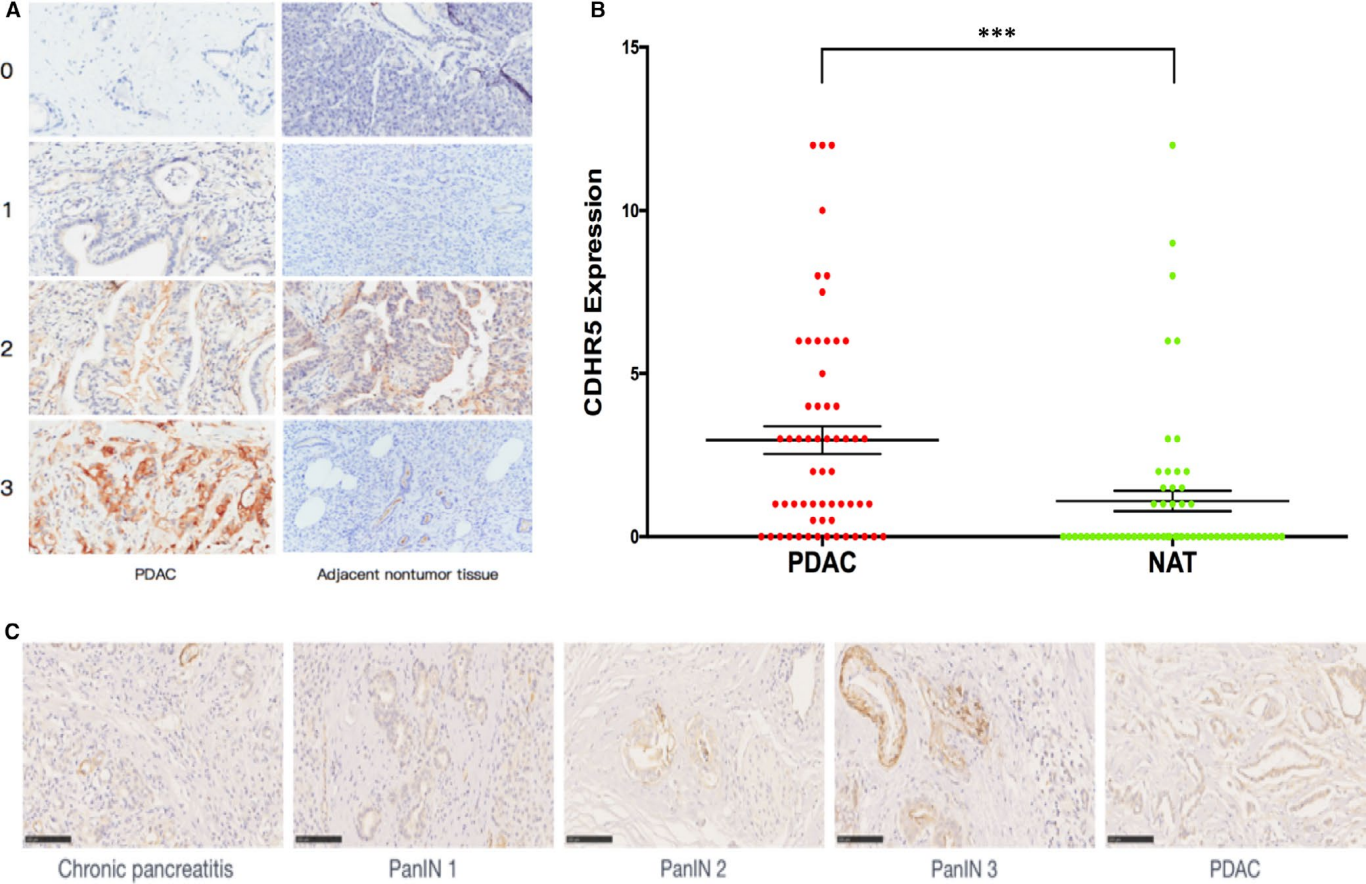
IHC staining of CDHR5 in PDAC and NAT tissues. A, The CDHR5 staining intensity was recorded as score 0‐3 in both PDAC and NAT samples. B, CDHR5 expression in PDAC and matched NAT was compared in each patient. C, Representative IHC staining pictures of CDHR5 expression in occurrence and progression of PDAC. Scale bar = 100 μm. ***P < .001

Fifty‐nine PDAC cases with both tumour and matched NAT samples were included in this analysis. Results of IHC staining showed that CDHR5 is mainly expressed on the membrane of PDAC cells, and expression was observed on the membrane of normal ductal cells (Figure 1A). Based on the IHC evaluation standards mentioned before, it is suggested that the expression of CDHR5 was remarkedly increased on the membrane of PDAC cells than that on the matched NAT (*P* < .001, Figure 1B). In addition, we have also analysed the prognostic value of CDHR5 in PDAC patients by using TCGA dataset. Results also showed that CDHR5 expression was significantly higher in pancreatic tumours compared to that in adjacent normal tissues (Figure S1).

### 3.2 Association of CDHR5 expression with clinicopathological features

To study the potential interaction between CDHR5 expression and clinicopathological features and prognosis of PDAC patients, sixty‐nine cases were finally enrolled in terms of the inclusive criteria. According to the optimal cut‐off value of CDHR5 expression, 40 (58.0%) patients were classified into the low expression group and 29 (42.0%) into the high expression group. No significantly association was observed between CDHR5 expression and main clinicopathological parameters (Table 1).

**Table 1:** CDHR5 expression and clinicopathological features of PDAC patients

Clinicopathological features	Cases	CDHR5 expression		*P* value
		Low	High	
Age (y)				
≥60	28	17	11	.703
<60	41	23	18	
Gender				
Male	43	25	18	.971
Female	26	15	11	
Tumour location				
Head	50	29	21	.994
Non‐head	19	11	8	
Pathological grade				
G1‐2	53	32	21	.461
G3	16	8	8	
T stage				
T1‐2	49	28	21	.827
T3‐4	20	12	8	
N stage				
N0	35	23	12	.151
N1‐2	33	16	17	
Nerve invasion				
Negative	43	25	18	.971
Positive	26	15	11	

### 3.3 Prognostic value of CDHR5 expression in PDAC patients

**Figure 2:**
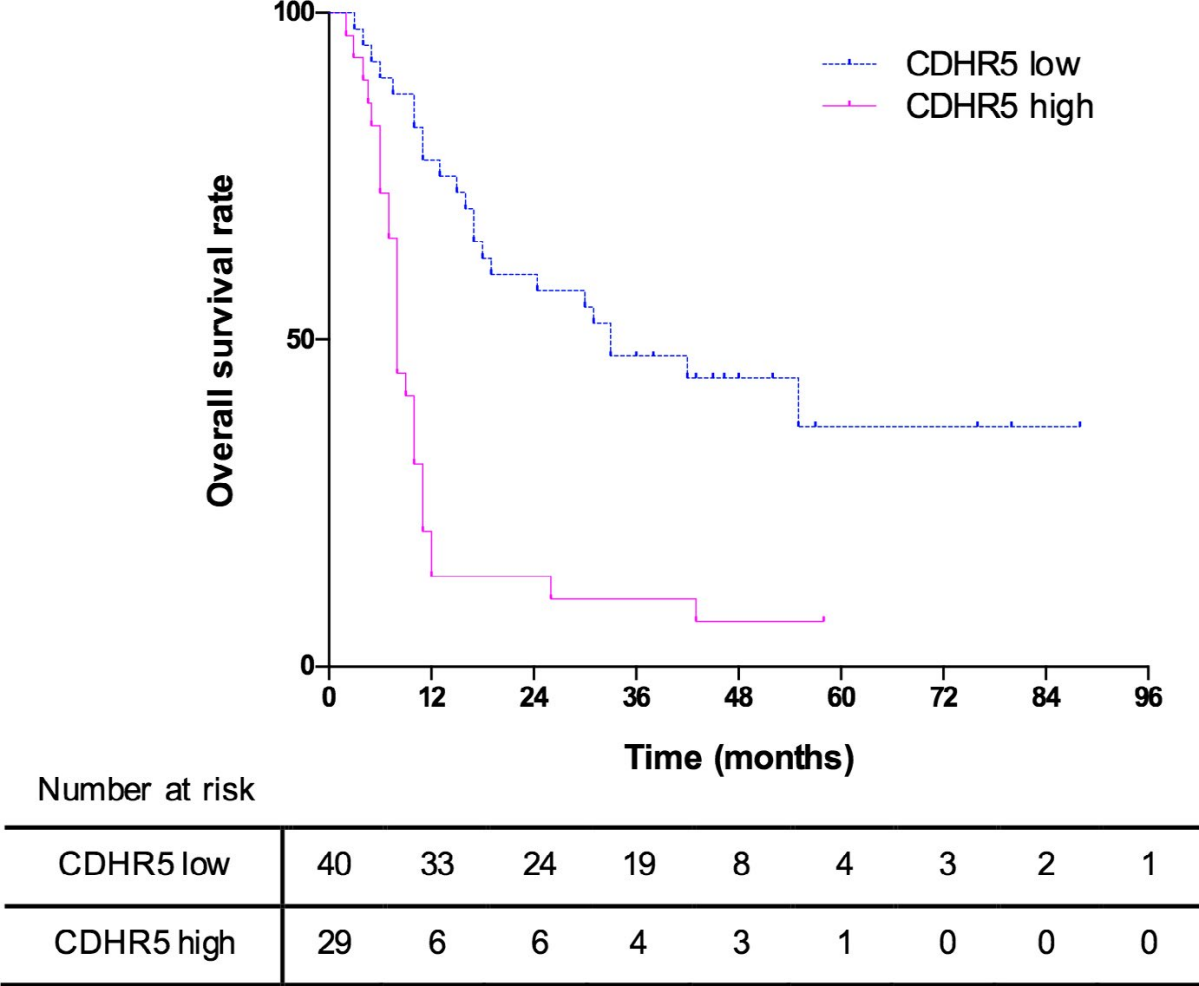
Kaplan‐Meier analysis of overall survival based on CDHR5 expression

Result of Kaplan‐Meier analysis suggested that PDAC patients in the CDHR5 high expression group had significantly shorter overall survival time (OS) than those patients with low expression of CDHR5 (median OS: 8 months vs 33 months, *P* < .001, Figure 2). In the univariate analyses, significance level was defined as *P* < .15 to avoid omitting important risk factors. As a result, pathological grade, N stage and CDHR5 expression were included for further multivariate Cox regression analysis, and results indicated that CDHR5 expression and pathological grade were independent prognostic factors for OS of PDAC patients (*P* < .001 and *P* = .014, respectively; Table 2).

**Table 2:** Univariate and Cox regression analyses for overall survival of PDAC patients

Clinicopathological features	Univariate analysis			Multivariate analysis		
	HR value	95% CI	*P*	HR value	95% CI	*P*
Age (y)						
≥60 vs <60	0.681	0.234‐1.978	.480			
Gender						
Male vs female	1.293	0.440‐3.801	.641			
Tumour location						
Head vs non‐head	1.607	0.457‐5.654	.460			
Pathological grade						
G3 vs G1‐2	3.306	0.674‐16.208	**.141**	0.432	0.221‐0.844	**.014**
T stage						
T3‐4 vs T1‐2	0.602	0.195‐1.857	.378			
N stage						
N1‐2 vs N0	2.659	0.868‐8.143	**.087**	0.696	0.378‐1.283	.245
Nerve invasion						
Positive vs negative	1.051	0.352‐3.135	.929			
CDHR5 expression						
High vs low	9.978	2.082‐47.826	**.004**	0.270	0.146‐0.501	**<.001**

### 3.4 Correlation of CDHR5 expression with tumour progression of PDAC

The CDHR5 IHC staining was performed in forty‐seven pancreatic tissues with different pathological grades, including 12 (25%) chronic pancreatitis (CP), 16 (34%) PanIN1, 10 (21%) PanIN2, 3 (7%) PanIN3 and 6 (13%) PDAC (Figure 1C). According to the optimal cut‐off value of CDHR5 expression, 27 (57.4%) patients were classified into the low expression group and 20 (42.6%) into the high expression group. Results showed that CDHR5 expression experienced a significantly increase with the occurrence and progression of PDAC (*P* = .028, Table 3). According to the updated 2‐tiered classification of neoplastic pre‐cursor pancreatic lesions, PanIN1 and PanIN2 were categorized as low‐grade PanIN (L‐PanIN), and PanIN3 was classified into the high‐grade PanIN (H‐PanIN).^11^ In this type of classification, chi‐square test also showed a significant difference of CDHR5 expression between CP＆L‐PanIN group and H‐PanIN＆PDAC group (*P* = .019, Table 4).

**Table 3:** CDHR5 expression in occurrence and progression of PDAC

Pathological diagnosis	CDHR5 expression		χ^2^	*P* value
	Low	High		
Chronic pancreatitis	11	1		
L‐PanIN	9	7	9.102	.028
H‐PanIN	5	8		
PDAC	2	4		
Total	27	20		

**Table 4:** CDHR5 expression of pancreatic tissues in different pathological grades

Pathological diagnosis	CDHR5 expression		χ^2^	*P* value
	Low	High		
Chronic pancreatitis & L‐PanIN	20	8	5.539	.019
H‐PanIN & PDAC	7	12		
Total	27	20		

### 3.5 CDHR5 promotes invasion and migration capacity of PDAC cells

**Figure 3:**
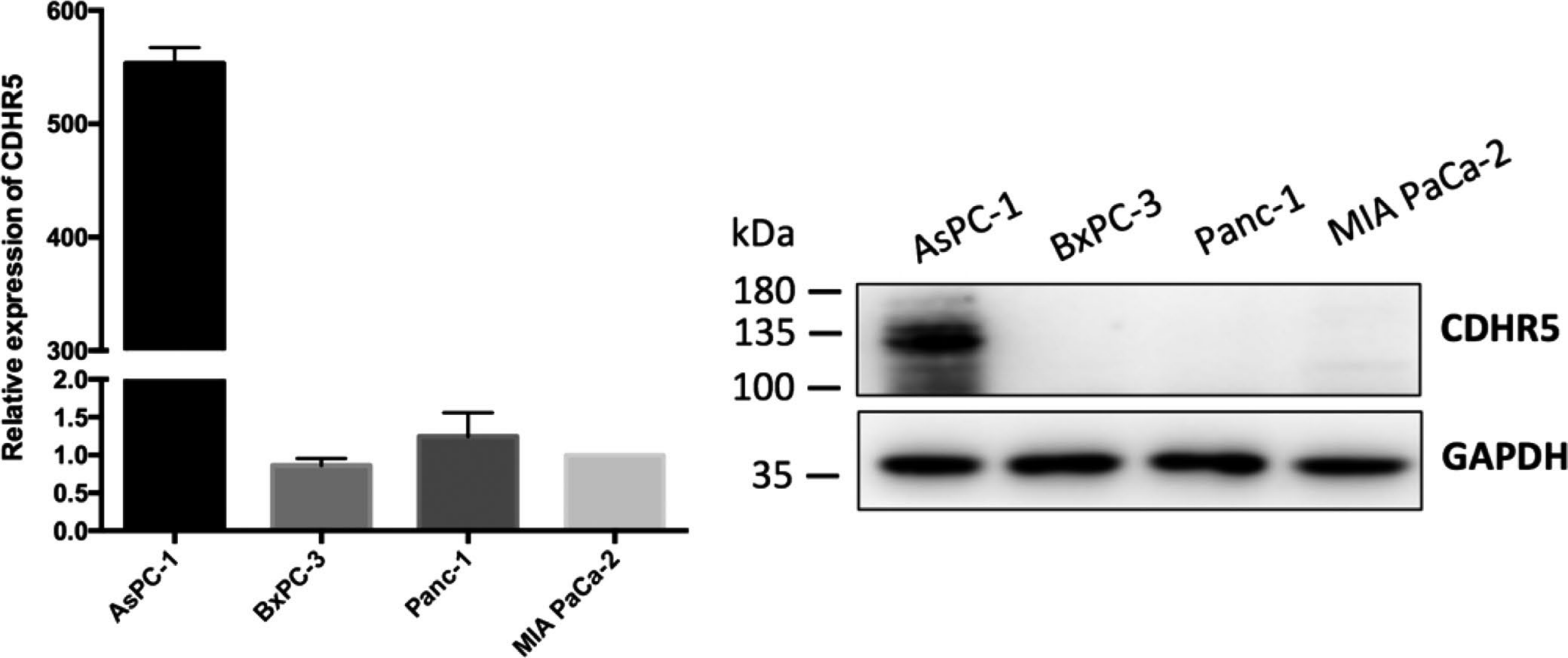
Expression of CDHR5 in PDAC cell lines. Relative expression of CDHR5 in mRNA level (left) and protein level (right)

**Figure 4:**
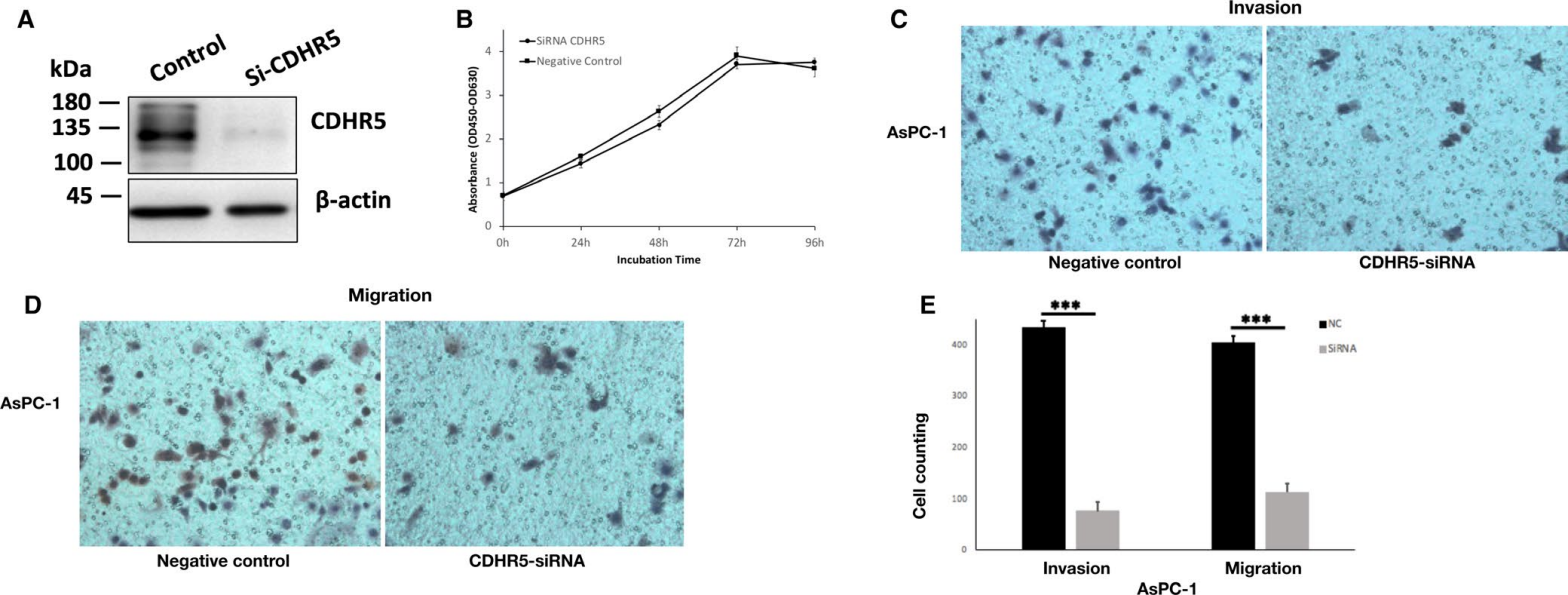
CDHR5 promotes invasion and migration capacity of PDAC cells. A, Knock‐down of CDHR5 expression by siRNA in AsPC‐1. B, Viability of AsPC‐1 cells treated with CDHR5‐siRNA was measured by CCK‐8 assay. C, Invasion capability of AsPC‐1 cells treated with CDHR5‐siRNA was measured by invasion assay. D, Migration capability of AsPC‐1 cells treated with CDHR5‐siRNA was measured by migration assay. E, Invasion and migration capability of AsPC‐1 cells treated with CDHR5‐siRNA. ***P < .001

**Figure 5:**
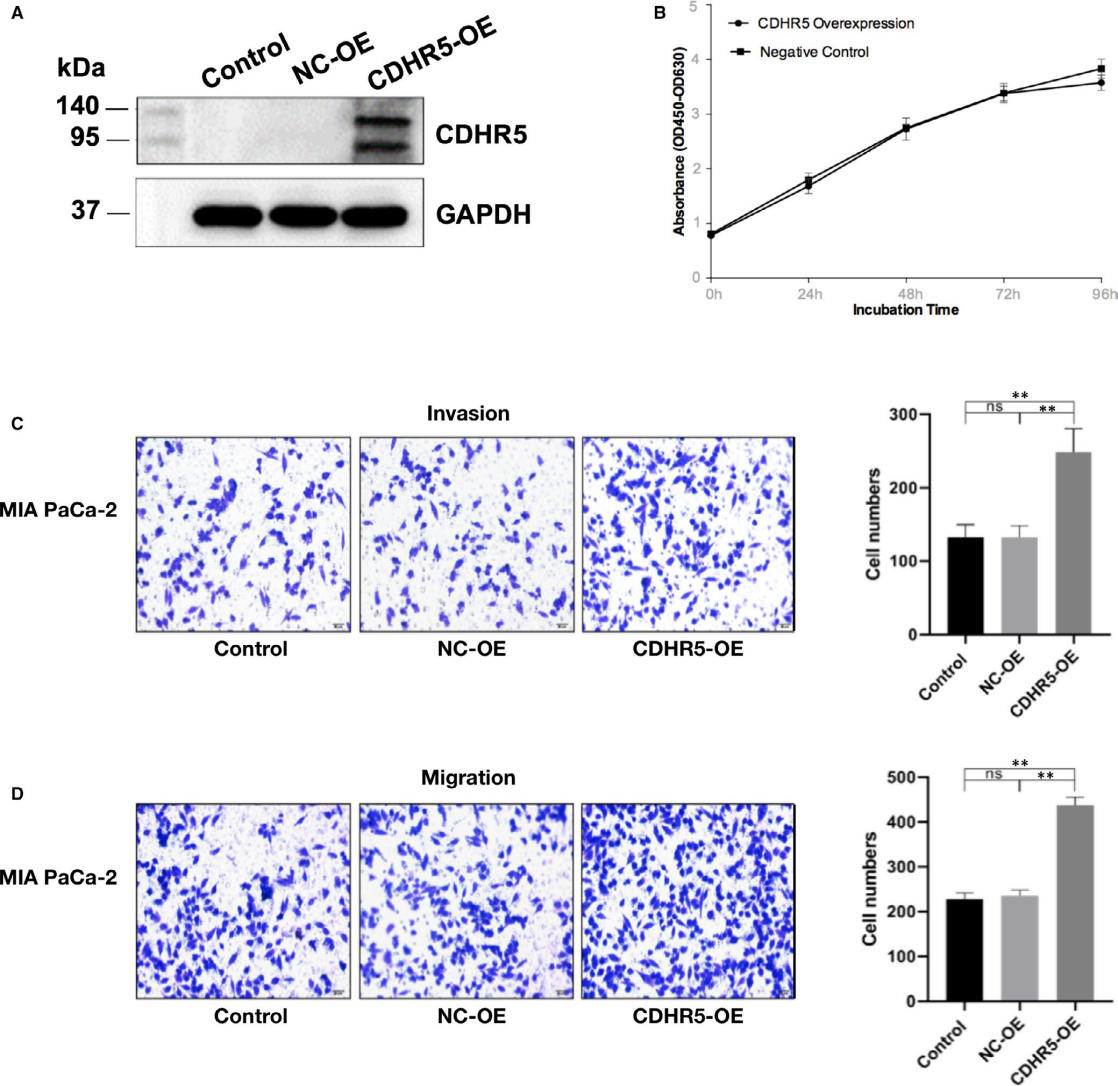
Overexpression of CDHR5 enhanced invasion and migration capacity of MIA PaCa‐2 cells. A, Overexpression of CDHR5 by plasmid vectors in MIA PaCa‐2. B, Effects of CDHR5 overexpression (CDHR5‐OE) on cell viability were measured by CCK‐8 assay. C, Invasion capability of MIA PaCa‐2 cells treated with CDHR5‐OE was measured by invasion assay. D, Migration capability of MIA PaCa‐2 cells treated with CDHR5‐OE was measured by migration assay. Scale bar = 20 μm. **P < .01

CDHR5 expression was evaluated in AsPC‐1, BXPC‐3, Panc‐1 and MIA PaCa‐2 cells both in mRNA and protein levels by qRT‐PCR and Western blot, respectively. Results of these two assays consistently showed that expression of CDHR5 extremely higher in AsPC‐1 cells than in the other three PDAC cell lines in both mRNA and protein levels (Figure 3). As a result, AsPC‐1 cell was determined to be used in the following cell function experiments. We firstly knocked down CDHR5 expression of AsPC‐1 cell via siRNA (Figure 4A). Results of CCK‐8 assay indicated that knock‐down of CDHR5 had no obvious effect on viability of AsPC‐1 cells (Figure 4B). However, the migration and invasion assay showed that down‐regulation of CDHR5 significantly suppressed the migration and invasion capability of AsPC‐1 cells (Figure 4C‐E). In addition, similar results were observed in the overexpression groups. It was showed that CDHR5 overexpression could not influence cell proliferation but remarkably promoted cell migration and invasion (Figure 5).

### 3.6 Expression of CDHR5 isoforms in PDAC

**Figure 6:**
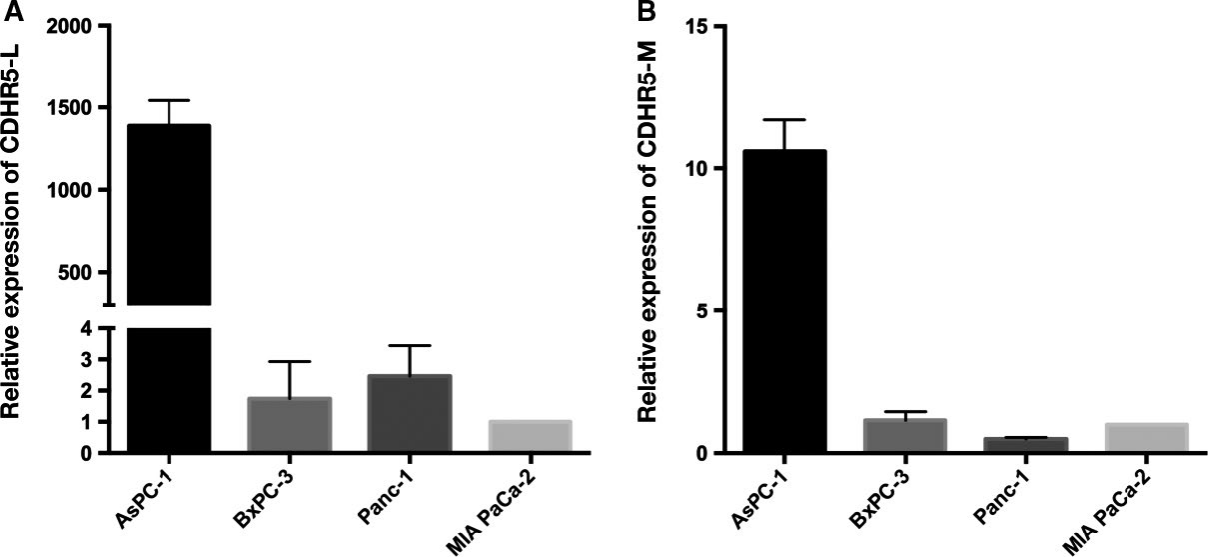
Expression of CDHR5 isoforms in PDAC cell lines. A, CDHR5‐L relative expression in PDAC cell lines. B, CDHR5‐M relative expression in PDAC cell lines

Expression of CDHR5 transmembrane isoforms, the CDHR5‐L and CDHR5‐M, was detected by qRT‐PCR, respectively, and results showed that expression of CDHR5‐L was extremely higher in AsPC‐1 cells than in the other three PDAC cell lines, while CDHR5‐M was about 10 times higher in AsPC‐1 cells than in the other three PDAC cell lines (Figure 6).

## 4 DISCUSSION

In the present study, we investigated the CDHR5 expression pattern and its clinical value in PDAC. Results showed that CDHR5 was extremely highly expressed in pancreatic tumour tissues and its high expression was associated with tumour metastasis. Moreover, the isoform CDHR5‐L was identified as the functional protein dominating in the process.

Studies of CDHR5 in renal cell carcinoma, colorectal cancers and liver cancer have consistently revealed low expression of CDHR5 on the tumour cell surface, and further mechanism researches disclosed that the dominating protein CDHR5‐M could retain β‐catenin on cell membrane via its PDZ domain, resulting in inhibition of Wnt/β‐catenin signalling pathway and down‐regulation of tumour proliferation.^12^, ^13^, ^14^

Felix et al have detected the CDHR5 expression in specimens of different renal cancer subtypes via IHC staining, and results showed that CDHR5 presented low expression level or even did not express in most of tumour samples. Additionally, most tumour samples with CDHR5 expression were histologically diagnosed as renal clear cell carcinoma and papillary renal cell carcinoma, while almost all chromophobe renal cell carcinoma were not detected with CDHR5 expression.^8^ In our study, we also analysis CDHR5 expression in pancreatic adenosquamous carcinoma in the TMA, outcomes suggested that CDHR5 was down‐regulated in this pancreatic cancer subtype, which was different from that in PDAC (data not shown). This result indicated that expression levels of CDHR5 were distinct in various tumours and in subtypes of the same tumour. Moreover, Felix's study also suggested the correlation between CDHR5 expression and pT stage of renal tumours, which could be explained as the outcomes of CDHR5‐M in tumour proliferation. However, no significant association was observed between CDHR5 expression and clinicopathological features of PDAC patients in our study. This phenomenon might be ascribed to the differentiation of CDHR5 functions in different tumours. In other words, CDHR5 might not remarkably affect tumour proliferation of PDAC while participate in metastasis process of tumour cells. As PDAC patients with tumour metastasis were excluded according to the criteria, the potential relation between CDHR5 and tumour metastasis was obscured inevitably. Thus, further cell experiment was needed to uncover more details.

Studies showed that CDHR5 expression fluctuated during organ development, and expression levels of different isoforms were regulated independently.^15^ Studies in colorectal cancer have also indicated that the expression level of CDHR5 mRNA in inflammatory or neoplastic intestinal mucosa was significantly lower than that in normal colon mucosa. Further IHC staining showed that CDHR5 expression in colon cancer also showed a decreasing tendency from low‐grade tumours to high‐grade tumours.^14^ Mechanism researches show that the expression levels of CDHR5 could be regulated by modulating transcript expression, promoter gene methylation, etc.^9^, ^16^, ^17^ However, our experimental results showed that the expression levels of CDHR5 showed an increasing trend during the progression of PDAC, which showed a markedly opposite change from the decreasing trend of CDHR5 expression in renal cancer and colorectal cancer. Considering that CDHR5 presents a high expression level in PDAC, this finding demonstrated that CDHR5 might play a role in promoting cancer in PDAC.

In cell experiments, Western blot and qRT‐PCR were applied to screen the CDHR5 expression levels in multiple PDAC cell lines. Results showed that the expression levels of CDHR5 in AsPC‐1 cell lines were significantly higher than that in the other three cell lines. Cell functional assays showed that down‐regulation of CDHR5 could extremely inhibit cell invasion and metastasis, while no effects were observed in the proliferation capacity. AsPC‐1 is a cell line derived from ascites metastasis of PDAC, and the BxPC‐3, Panc‐1 and MIA PaCa‐2 cell lines are all derived from PDAC in situ. Hinkel et al have primarily studied the functions of CDHR5 isoforms, and it was showed that CDHR5‐M could significantly induce apoptosis of colon cancer cells, while CDHR5‐L has a weaker effect on apoptosis, and the results in different cell lines vary greatly.^9^ This result indicated that the CDHR5 isoform that played a leading role in PDAC might not be CDHR5‐M, but other CDHR5 isoforms that were highly expressed in metastatic pancreatic cancer. At present, some evidence has been found that could indicate the CDHR5 effects in cell movement. Firstly, the two transmembrane isoforms, CDHR5‐L and CDHR5‐M, contained PDZ domains in their intracellular segments, which could affect cell morphology and movement via connecting to the actin cytoskeleton.^18^ Secondly, the mucin domain exists in CDHR5 could promote the rolling movement of lymphocytes by linked to L‐selectin.^19^ Thirdly, a variety of cadherins has been shown to participate in the construction of the three‐dimensional structure of organs, and this process is also accompanied by changes in cell morphology and cell movement; for instance, cadherin 6, cadherin 11 and E‐cadherin are all involved in the formation of renal tubules.^20^ Furthermore, in vivo experiments also showed that CDHR5 presented a distribution preference on the cell membrane, this suggested that glycosylated CDHR5 could mediate cell polarization, and its overexpression might lead to loss of cell polarity,^21^ inducing transformation to interstitial or stem cell, resulting in tumour metastasis through epithelial mesenchymal transformation.^22^

Detection of CDHR5 isoforms showed that the expression level of CDHR5‐L in AsPC‐1 cells was significantly higher than that in the other cell lines, while the CDHR5‐M expression difference was relatively insignificant among the cell lines. Based on the outcomes achieved above, it could be predicted that CDHR5‐L might play a key role in metastasis of PDAC. The structure difference between CDHR5‐L and CDHR5‐M is that the former has a unique mucin domain. However, the functional research of CDHR5‐L is very limited. Further researches are needed to clarify the exact functions of CHDR5‐L and the mucin domain. In addition, the widespread of CDHR5 in many normal tissues such as kidney and lung would limit the application of drugs targeting CDHR5. As a result, the potential CDHR5‐based drugs would be applied via targeting drug delivery system to avoid possible harmful effects on other organs and tissues in the future. Additionally, present studies have suggested that CDHR5‐L was the major isoform regulating malignant behaviours of PDAC, while CDHR5‐M was dominantly expressed in other cancers, so specific sites of CDHR5‐L like the mucin domain would be designed as the therapeutic targets to prevent adverse effects on other organs and tissues.

## 5 CONCLUSION

In conclusion, our study revealed that CDHR5 expression was correlated with outcomes of PDAC patients and could be a promising prognostic factor for PDAC. As the isoform CDHR5‐L might function in promoting PDAC metastasis, it provided a novel therapeutic target for PDAC management.

## CONFLICT OF INTERESTS

The authors declare that they have no competing interests.

## AUTHOR CONTRIBUTIONS

**Junyi Gao:** Conceptualization (lead); Data curation (lead); Formal analysis (lead); Investigation (lead); Methodology (lead); Resources (equal); Writing‐original draft (lead); Writing‐review & editing (lead). **Mengyi Wang:** Data curation (supporting); Investigation (equal); Methodology (equal); Project administration (equal); Writing‐review & editing (equal). **Tong Li:** Formal analysis (supporting); Investigation (supporting); Methodology (supporting); Writing‐review & editing (equal). **Qiaofei Liu:** Formal analysis (supporting); Investigation (supporting); Methodology (supporting); Writing‐review & editing (supporting). **Lei You:** Project administration (equal); Resources (equal); Supervision (equal); Writing‐review & editing (equal). **Quan Liao:** Funding acquisition (lead); Investigation (equal); Project administration (lead); Resources (lead); Supervision (lead); Writing‐review & editing (equal).

## CONSENT FOR PUBLICATION

Not applicable.

## Supporting information

Fig S1

## Data Availability

The data sets used and/or analysed during the current study are available from the corresponding author on reasonable request.
