# Supplementary material for: Up‐regulation of CDHR5 expression promotes malignant phenotype of pancreatic ductal adenocarcinoma
Source: J Cell Mol Med. 2020 Oct 6;24(21):12726–35. doi: 10.1111/jcmm.15856 (PMC7687006; doi:10.1111/jcmm.15856)
Supplement: Supplementary file 1 — Fig S1 [file JCMM-24-12726-s001.docx]

**Supplementary** **Figure legends**

**
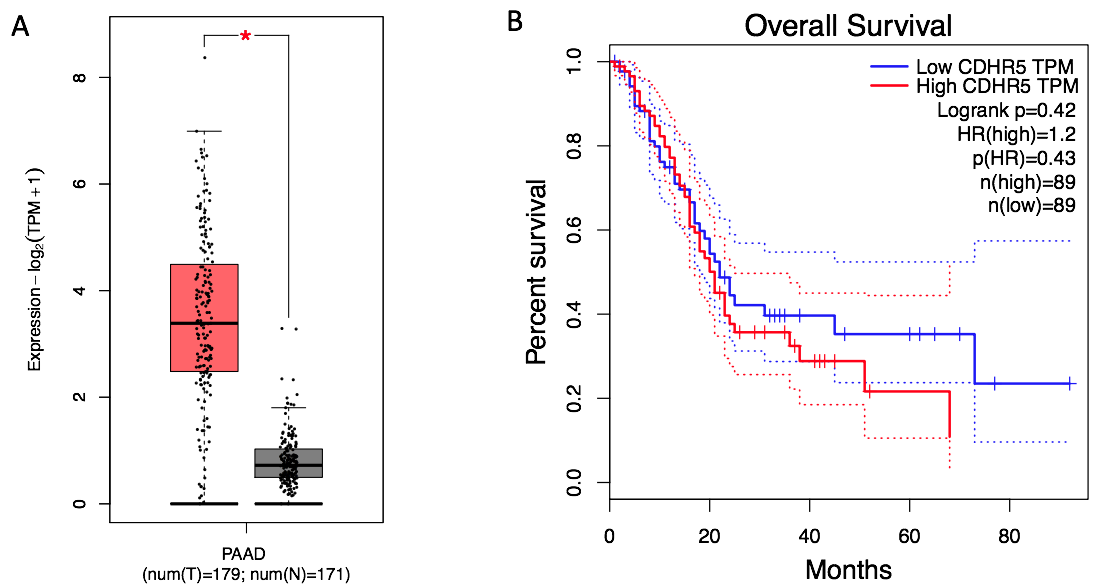
**

**Supplementary Figure S1. Expression of CDHR5 in PDAC and NAT tissues, using the TCGA dataset.** (A) CDHR5 expression was remarkably higher in PDAC tissues compared to that in NAT tissues (*p<0.05). (B) Survival analysis showed no significant difference between the CDHR5 high expression and low expression groups.
